# Non-muscle myosins control the integrity of cortical radial glial endfeet

**DOI:** 10.1371/journal.pbio.3002032

**Published:** 2023-02-28

**Authors:** Li Wang, Arnold R. Kriegstein

**Affiliations:** 1 The Eli and Edythe Broad Center of Regeneration Medicine and Stem Cell Research, University of California, San Francisco, San Francisco, California, United States of America; 2 Department of Neurology, University of California, San Francisco, San Francisco, California, United States of America

## Abstract

Radial glial cells, the stem cells of the cerebral cortex, extend a long basal fiber that ends in basal endfeet. This Primer explores the implications of a new PLOS Biology study revealing that non-muscle myosins control basal endfoot integrity to regulate interneuron organization.

## Teaser

Radial glial cells, the stem cells of the cerebral cortex, extend a long basal fiber that ends in basal endfeet. A new study in *PLOS Biology* found that non-muscle myosins control basal endfoot integrity to regulate interneuron organization.

**Fig 1:**
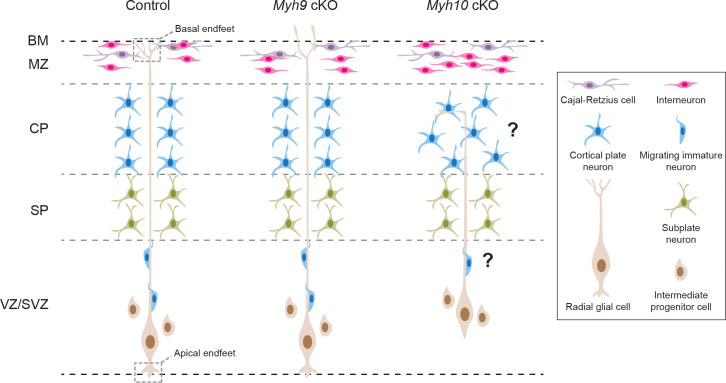
Non-muscle myosins MYH9 and MYH10 control RGC basal endfoot integrity to regulate interneuron organization. In *Myh9* cKO cortex, RGCs are less complex basally and have fewer basal endfeet; the basal endfeet protrude abnormally through the basement membrane; interneurons detach from the basement membrane. In *Myh10* cKO cortex, RGCs gradually lose apical and basal endfoot attachment; the number of interneurons in the marginal zone increases by ~40%. It would be interesting to determine if migration and organization of cortical plate neurons are also affected by endfoot detachment. BM, basement membrane; cKO, conditional knockout; CP, cortical plate; MZ, marginal zone; RGC, radial glial cell; SP, subplate; VZ/SVZ, ventricular zone/subventricular zone.

Radial glial cells (RGCs) are neural stem cells in the developing cerebral cortex responsible for producing neurons and glia. They have an elongated bipolar morphology that spans the thickness of the developing cortex. RGC somata are located in the ventricular zone and connect to the ventricle through apical endfeet (Fig 1). On the basal side, they extend a long basal fiber that contacts the basement membrane in the pia matter through basal endfeet (Fig 1). Basal endfeet are embedded in a unique niche between the marginal zone and the pia. They receive a wealth of extrinsic cues, including retinoic acid and growth factors that regulate RGC proliferation [1]. Conversely, the basal fibers and endfeet provide physical and molecular guidance for neuronal migration [2,3]. However, the molecular composition of these basal structures and the mechanisms by which they are regulated are largely unknown.

In this issue of *PLOS Biology*, D’Arcy and colleagues determined the proteomic composition of basal endfeet in mice through a combination of microdissection and proximity proteomics [4]. They identified 47 proteins that are abundant and enriched in basal endfeet relative to the rest of the RGC. These include extracellular matrix proteins, microtubule-associated proteins, and actomyosin components. Non-muscle myosin II heavy chain isoforms, including MYH9 and MYH10, were among the most enriched proteins from the mouse endfoot proteome. Interestingly, transcripts of *Myh9* and *Myh10* are localized to basal endfeet with distinct expression patterns, suggesting that they are locally translated and may serve different functional roles during development.

To understand the possible importance of MYH9 and MYH10 for endfoot morphology and function, the authors generated conditional knockout (cKO) mice to remove MYH9 or MYH10 from RGCs. They found that *Myh9* cKO RGCs had fewer and less complex endfeet that protruded abnormally through the basement membrane into the pia (Fig 1). In contrast, *Myh10* cKO RGCs gradually lost apical and basal endfoot attachment during late neurogenesis, leading to their displacement from the ventricle (Fig 1). The divergent phenotypes of *Myh9* and *Myh10* cKO RGCs highlight the distinct functions of each non-muscle myosin isoform in controlling endfoot morphology and RGC integrity.

Do the abnormal endfeet induced by *Myh9* and *Myh10* cKO impact surrounding cells? The authors examined the general architecture of the marginal zone, which, among other functions, serves as one of the major routes for interneuron migration to the cortex. They found LHX6+ interneurons detached from the basement membrane in both *Myh9* and *Myh10* cKO mice (Fig 1). In addition, in the *Myh10* cKO cortex, where basal endfeet detached from the basement membrane, there was an approximately 40% increase in LHX6+ interneurons in the marginal zone (Fig 1). Together, these results demonstrate that RGC endfeet are crucial for both interneuron organization and number in the marginal zone of the developing cortex.

This study uncovered the proteomic composition of RGC basal endfeet and identified that non-muscle myosins are not only enriched in the endfeet but are also required to maintain RGC integrity. One question that naturally arises is how MYH9 and MYH10 regulate RGC endfeet position and attachment, respectively. Non-muscle myosins can inhibit cellular protrusions by modulating actin dynamics [5]. Thus, MYH9 may limit basal endfeet from crossing the basement membrane through a similar mechanism. Adhesion molecules, including integrins, are required for the attachment of RGC endfeet to the basement membrane [6]. MYH10 could be required for the interaction between actomyosin and adhesion molecules to promote endfoot attachment. Future work is warranted to determine if these potential mechanisms hold true. Interestingly, RGC endfeet normally detach toward the end of cortical development as part of the transformation of RGCs into astrocytes [7]. One fascinating question would be whether non-muscle myosins are also involved in this process and whether their removal accelerates the conversion of RGCs to astrocytes.

D’Arcy and colleagues highlight the crucial role of RGC basal endfeet for interneuron organization in the marginal zone and point to developmental defects upon disruption of the endfeet. These findings are consistent with previous human genetic studies indicating that both *MYH9* and *MYH10* are associated with neurological disorders including microcephaly and developmental delay [8,9]. In the future, it would be important to determine whether and how RGC endfoot dysfunction caused by these mutations contributes to later neurological phenotypes. This could be done by investigating the long-term impact of *Myh9* and *Myh10* RGC cKO in mouse cortex on cortical organization. Given the importance of radial fibers and endfeet of RGCs for neuronal migration, the allocation of excitatory and inhibitory neurons in the mature cortex may also be affected in the cKO mice. Indeed, the increase in the number of interneurons in the marginal zone of *Myh10* cKO mice suggests a potential disruption of interneuron radial migration from the marginal zone to the cortical plate, highlighting the possible existence of more extensive cortical migration defects.
